# Biodiversity research sets sail: showcasing the diversity of marine life

**DOI:** 10.1098/rsbl.2008.0735

**Published:** 2009-01-07

**Authors:** Thomas J. Webb

**Affiliations:** Department of Animal and Plant Sciences, University of SheffieldSheffield S10 2TN, UK

**Keywords:** marine biodiversity, conservation, taxonomy, science–policy interface

## Abstract

The World Congress on Marine Biodiversity was held in the City of Arts and Sciences, Valencia, from 10 to 15 November 2008, showcasing research on all aspects of marine biodiversity from basic taxonomic exploration to innovative conservation strategies and methods to integrate research into environmental policy.

## 1. Introduction

A 2006 *Science* editorial complained that biodiversity research remained ‘grounded’ (Hendriks *et al*. 2006): only approximately 10 per cent of the research published or presented at international biodiversity conferences is marine, a similar proportion to that found in related disciplines including conservation biology and macroecology (Raffaelli *et al*. 2005; Richardson & Poloczanska 2008). In addition, much of that 10 per cent is published in marine journals that fail to reach the ecological ‘mainstream’ (Raffaelli *et al*. 2005)—the large community of mainly terrestrial ecologists, which dominates discussions with policy makers and the wider public. As a consequence, many people remain largely unaware of the extent of marine biodiversity, with clear consequences for policy. For instance, the 43 members of the Intergovernmental Panel on Climate Change working group investigating the effects of climate change on ecosystems included only four marine specialists; the data they examined were overwhelmingly (95%) terrestrial; and only a tiny fraction (0.3%) of the 29 000 systems in which they found significant biological changes were marine (Richardson & Poloczanska 2008).

Chaired by two of the authors of the *Science* editorial (Carlos Duarte and Carlo Heip), the World Congress on Marine Biodiversity marked a clear effort to address this imbalance, by raising the profile of marine biodiversity research and the networks that support it. The response of the community suggests a thriving discipline: 510 delegates from 42 countries were matched by an equal number who would have attended had space allowed. Over 5 days there were 280 presentations, 160 posters and 9 plenaries, in addition to a policy round-table and a series of public engagement events.

As the meeting progressed, however, the state of marine biodiversity research was revealed to be rather more complex. A wealth of talks on basic taxonomic exploration emphasized that increased sampling of the sea simply reinforces that we still have little idea what's actually out there; yet at the same time there was a general consensus (formalized as the Valencia Declaration, http://tinyurl.com/5kv852) that marine systems are in crisis, and require urgent and innovative conservation action. The paradox then is to try to value and conserve taxa and habitats that remain largely unknown. One way to link the inventorying (What's out there?) and the crisis management (What are we doing to marine biodiversity?) is to address more fundamental ecological questions concerning the functioning of marine ecosystems (What does marine biodiversity do?). Much of the conference can usefully be summarized under these broad questions.

## 2. What's out there?

In his opening plenary, Carlo Heip (Netherlands Institute of Ecology/Royal Netherlands Institute for Sea Research) observed that the discovery of new marine species shows no signs of slowing down. This simple observation proved a common theme across many contributions, but what may surprise terrestrial ecologists is the degree of uncertainty in the estimates of the number of marine species even in relatively well-known areas. For example, up to a quarter of European marine species may remain to be discovered (Wilson & Costello 2005); Mark Costello (University of Auckland) suggested that this figure might rise to 33–92% in less intensively studied parts of the world, such as the tropics. Philippe Bouchet (Muse´um National d'Histoire Naturelle, Paris) wryly noted that, while ecologists and conservationists often focus on ‘megadiverse’ ecosystems, they are also very good at carefully avoiding the most ‘difficult’ (but often most diverse) taxa such as molluscs, crustaceans and polychaetes. By focusing explicitly on such groups, Bouchet's expeditions have found more species of mollusc in 3000 New Caledonian hectares than that occur in the entire Mediterranean. Importantly, the real diversity lies in rare, difficult to sample and hard to identify species—up to a third of which are too small to be retained in standard sampling gear. Expeditions to other undersampled habitats have produced similar findings: Steven Haddock (Monterey Bay Aquarium Research Institute) reported that recently discovered ctenophores (comb jellies) from the deep pelagic ocean (a region that constitutes more than 90% of the Earth's habitable volume) are likely to represent previously unknown lineages, constituting new branches rather than terminal twigs on the phylogenetic tree.

Such expeditions produce many thousands of animals that require identification, and a common lament was that a shortage of expert taxonomists is hindering description of marine biodiversity. For instance, only approximately 100 out of more than 5000 unknown marine animals discovered by the Census of Marine Life (www.coml.org) in the last 5 years have been officially described. As Costello noted, this description needs to speed up if a full inventory of marine species is ever to be reached. One approach (championed by Bouchet) is to throw people at the problem and create taxonomic production lines in the field. New technologies are also helping, in particular DNA barcoding. Ann Bucklin's (University of Connecticut) ship-based studies employ on-board DNA sequencing to rapidly assign individuals to species using a library of DNA barcodes for some 7000 species of deep-sea zooplankton. Extending this approach to bulk processing of entire communities will help the taxonomic effort by allowing expert taxonomists to concentrate on those individuals that do not match any known barcodes.

## 3. What does it do?

This uncertainty about what exists in the seas should not stop efforts to understand marine ecology—after all, great strides have been made in terrestrial ecology without us knowing every species of beetle in Amazonia. This meeting provided a flavour of the significant ecological contributions made by marine scientists. Marine systems provide unique challenges to, and tests of, ecological theory, perhaps most strikingly the existence of so many coexisting species in what appear to be large, homogeneous habitats. However, even the pelagic realm can be partitioned at surprisingly fine scales. Holger Auel (University of Bremen) showed how deep sea copepods partition their habitat by depth, and more generally Gregory Beaugrand (National Centre for Scientific Research) used long-term, large-scale data to reveal how temperature can delimit North Atlantic plankton communities. Microhabitats can be very important too. For instance, the species composition on the blades of kelp can be very different from that on the holdfasts of the same plants; Christie Hartvig (Norwegian Institute for Water Research) showed how dietary specialization in fish feeding on these invertebrates in turn leads to fine-scale habitat associations of fishes. Jeff Huismann (University of Amsterdam) has combined experimental and theoretical approaches to reveal the chaotic dynamics driving coexistence in plankton populations, but these complex dynamics mean that predictions of future dynamics (much like weather forecasts) will be accurate only in the short term. This highlights the need for continuous monitoring of marine populations, which Steve Hawkins (Bangor University) showed is also essential for understanding and predicting ecological effects of climate change.

The functional consequences of marine diversity were examined in terms of the relationship between biodiversity and ecosystem functioning. As Emmett Duffy (Virginia Institute of Marine Science) showed, experiments have revealed that systems with higher species diversity in general tend to have higher ‘function’ (primary production, nutrient cycling, trophic transfer, etc.); he suggests that these patterns are also seen for example in the functional consequences of global fisheries declines (Worm *et al*. 2006). Many of the other talks on this theme focused on the intricacies of this general pattern, including Mark Bulling's (University of Aberdeen) demonstration that ecosystem functioning can be influenced by complex interactions between CO_2_ and temperature as well as species richness and identity.

## 4. What are we doing to it?

The Valencia declaration is entitled ‘A Plea for the Protection of Marine Biodiversity’, encapsulating well the major theme of the conference: human activity is pervasive and increasing in the marine environment, and even if we cannot yet fully inventory marine diversity, we know enough to fear for its future. Predicting this future is difficult, of course: Simonetta Fraschetti (Laboratory of Zoology and Marine Biology, Italy) emphasized that we can *map* human activities, but we seldom know what their *effects* will be upon biodiversity. One activity with stark ecological effects, however, is what Daniel Pauly (University of British Columbia) termed ‘the global fisheries machine’. According to Pauly, this machine has stopped working: the international fishing fleet continues to expand its energy use (fisheries already produce 1% of anthropogenic carbon emissions) and the spatial and taxonomic scale of its efforts, but landings are declining. So far imports from poorer nations have hidden this fact from consumers in the North, but Heike Lotze (Dalhousie University) showed how using historical records (for instance mediaeval paintings of fish markets) can reveal profound changes in fish communities, which may lead to cascading effects through the ecosystem.

Pauly emphasized the failure of traditional methods of managing fisheries, for instance applying a simple optimization paradigm to complex multi-species, multi-user systems where no optimum exists—instead, management must involve compromise. According to Sybille Van den Hove (MEDIAN), ecologists must adapt by working with economists and social scientists: simply doing more ecology is insufficient. A complementary approach, outlined by Juan Carlos Castilla (Pontificia Universidad Católica de Chile), is for management to be driven by user needs. He described schemes in Chile that give fishers themselves exclusive rights to small areas, to encourage effective management. The success of such schemes (Gelcich *et al*. 2008) demonstrates the importance of engaging people. As Castilla puts it, ‘There is no silver bullet, but there are silver principles’. One of these may be to protect more of the sea from human activities. Mike Kaiser (Bangor University) presented evidence that, in general, marine protected areas (MPAs) have measurable (if rather variable) effects on diversity and biomass; Carlos Duarte (CSIC-UIB) reminded us that MPAs remain an underused tool, and at present cover only approximately 10 per cent the area protected on land.

## 5. So what?

Daniel Pauly urged us to have a ready answer to the ‘So what?’ question beloved of politicians and journalists: why should we care about the extent of, and threats to, marine biodiversity? The simplest answers are utilitarian, including the potential of natural products to inspire new drugs. Raymond Andersen (University of British Columbia) presented a simple equation: more biodiversity means more chemical diversity. Dolph de Groot (Wageningen University) expanded this idea to outline more generally the ‘ecosystem services’ on which we depend, including global marine fisheries, carbon sequestration and coastal protection, and the cultural and aesthetic value we derive from the marine environment. Entranced by the fishes in the enormous aquarium backing the main lecture theatre, it was impossible to disagree that marine diversity can contribute to human well-being! Yet approximately 60 per cent of marine services are degraded, at a cost to society that de Groot reckoned dwarfs that of the current economic crisis. Market failures have resulted from a mismatch between the private benefits and public costs of degrading marine environments, but Mel Austin (PML) stressed that money is the universal language of politics. She showed how putting specific values on ecosystem services raises awareness of the importance of marine biodiversity (Beaumont *et al*. 2008), but also acknowledged that we lack data for comprehensive valuation even in a well-studied area (the Scilly Isles) of a well-studied country (the UK).

## 6. Prognosis

The World Congress on Marine Biodiversity was successful in publicizing the vast amount of research that is conducted into the biological diversity of the seas, and it was understandable that the uniqueness of marine systems was often emphasized. However, recognizing that marine–terrestrial differences are not as clear-cut as sometimes assumed would help increase the profile of marine biodiversity research both in international policy and in the published scientific record. One series of talks was dedicated to John Gray, the prolific biodiversity scientist who died in 2008. Gray recognized that differences between marine and terrestrial systems could result from different sampling regimes (Gray *et al*. 2006): sample in similar ways, and you observe similar patterns. More recently, Dawson & Hamner (2008) have shown that it is easy to overemphasize even the physical differences between the land and the sea, and that rather than drawing a simple marine–terrestrial division, recognizing the continuum of environments within both realms can make for more interesting comparisons. Testing theory derived from terrestrial studies using data from taxonomically and functionally diverse marine systems (e.g. Webb *et al*. in press) can advance biodiversity research across systems.

Protecting marine diversity raises similar issues: the seas pose unique management problems (especially in terms of jurisdiction), but many others are common to all systems (e.g. conflicts between private profits and public costs, problems of multiple human activities coexisting in space). Alyne Delaney (Roskilde University), who has been investigating the socio-cultural valuation of marine biodiversity on the Scilly Isles, found that the islanders did not differentiate between marine and terrestrial biodiversity. Were this intriguing finding to generalize, it would provide a strong impetus for the biodiversity research community to speak with one voice across environments. The new Intergovernmental Platform on Biodiversity and Ecosystem Services (http://ipbes.net) could provide a forum for this. Biodiversity research needs to set sail, but the ship will progress best if it remains in communication with the land.
